# Adherence to Referral Criteria at Admission and Patient Management at a Specialized Burns Centre: The Case of the Red Cross War Memorial Children’s Hospital in Cape Town, South Africa

**DOI:** 10.3390/ijerph14070732

**Published:** 2017-07-06

**Authors:** Constance Boissin, Marie Hasselberg, Emil Kronblad, So-Mang Kim, Lee Wallis, Heinz Rode, Lucie Laflamme

**Affiliations:** 1 Department of Public Health Sciences, Karolinska Institutet, Tomtebodavagen 18A, 171 77 Stockholm, Sweden; marie.hasselberg@ki.se (M.H.); kronblad@gmail.com (E.K.); lucie.laflamme@ki.se (L.L.); 2 Faculty of Health Sciences, University of Cape Town, Anzio Road, Observatory, Cape Town 7935, South Africa; kmxsom002@myuct.ac.za (S.-M.K.); heinz.rode@uct.ac.za (H.R.); 3 Division of Emergency Medicine, Faculty of Medicine and Health Sciences, Stellenbosch University, Private Bag X24, Bellville 7535, South Africa; leew@sun.ac.za; 4 Division of Emergency Medicine, Faculty of Health Sciences, University of Cape Town, Private Bag X24, Bellville 7535, South Africa; 5 Department of Paediatric Surgery, Red Cross War Memorial Children’s Hospital, Cape Town 7700, South Africa; 6 Institute for Social and Health Sciences, University of South Africa, P.O. Box 1087, Lenasia 1820, South Africa

**Keywords:** triage, referral, paediatrics, resource-poor setting, injury

## Abstract

Referral guidelines for burn care are meant to assist in decision-making as regards patient transfer and admissions to specialized units. Little is known, however, concerning how closely they are followed and whether they are linked to patient care. This is the object of the current study, focused on the paediatric burns centre of the Red Cross War Memorial Children’s Hospital in Cape Town, South Africa. All patients admitted to the centre during the winters of 2011–2015 (*n* = 1165) were included. The patient files were scrutinized to clarify whether the referral criteria in place were identified (seven in total) and to compile data on patient and injury characteristics. A case was defined as adherent to the criteria when at least one criterion was fulfilled and adherence was expressed as a percentage with 95% confidence intervals, for all years aggregated as well as by year and by patient or injury characteristics. The association between adherence to any individual criterion and hospital care (surgery or longer length of stay) was measured using logistic regressions. The overall adherence was 93.4% (100% among children under 2 years of age and 86% among the others) and it did not vary remarkably over time. The two criteria of “injury sustained at a specific anatomical site” (85.2%) and “young age” (51.9%) were those most often identified. Children aged 2 years or older were more likely to undergo surgery or to stay longer than those of young age (although a referral criterion) and so were those with higher injury severity (a referral criterion). In this specialized paediatric burns centre, children are admitted mainly according to the guidelines. However, given the high prevalence of paediatric burns in the region and the limited resources at the burns centre, adherence to the guidelines need to be further studied at all healthcare levels in the province.

## 1. Introduction

Burns are the third most common cause of injury-related deaths worldwide for children under the age of 18 years [1]. Indeed, 30% of those who die from burns are under 20 years of age, and infants have the highest burn-related death rates [1]. In addition, almost 95% of burn-related deaths occur in low- and middle-income countries (LMICs) [2], where healthcare systems are already overburdened due to fighting communicable diseases and malnutrition in addition to being poorly prepared to deal with acute trauma [3].

In the Sub-Saharan region, burn injuries represent a challenge to clinical practitioners due to their high prevalence [4], the lack of key specialized resources and services like dedicated burns centres [5], and the difficulties faced by front line practitioners in making an accurate diagnosis, particularly concerning the wound depth and size [6,7]. Yet, decisions such as type of dressing, need for fluid resuscitation, indication for surgical treatment, and transfer to a burns centre are all based on the initial diagnosis. Inaccurate burns assessments may lead to under- and over-triage: the former largely affects burn morbidity and mortality and the latter may lead to unnecessary referral and treatment, causing unnecessary pressure on already limited human and material resources such as beds [8].

One way to deal with burn referral issues is the implementation of context-appropriate referral criteria. Although such guidelines have been established in a number of countries [9,10,11], including South Africa, where this study was conducted [12], neither their implementation nor their impact on healthcare services have been much studied. However, recent studies have shown that there is a high number of patients that could not be transferred from lower levels of care to the burns centre [13,14]. It is unclear whether this is a question of volume only, or whether there may be issues in adherence to the referral criteria upon admission to the burns centre. The latter question therefore deserves particular attention in order to understand if, at the end of the referral chain, patients who are admitted should be, according to the criteria. The primary aim of this study is to determine at the Red Cross War Memorial Children’s hospital’s burns centre, in South Africa, the level of adherence to the provincial referral criteria, considering potential changes over time and by patient and injury characteristics. A secondary aim of this study is to assess which of the specific referral criteria are associated with the care received at the centre.

## 2. Materials and Methods

### 2.1. Study Design

A medical records-based study was conducted encompassing acute burn patients admitted to the burns centre at the Red Cross War Memorial Children’s Hospital (RCH) in Cape Town, South Africa during five consecutive winter seasons (1 May–15 August; 2011–2015).

### 2.2. Setting

The Western Cape Province has been described as a prototype of the African continent with regard to paediatric burn injuries due to its high burn-related morbidity, and to the demographic and living characteristics of the region contributing to a high burden [15]. Indeed, the province has a rapidly increasing population with almost 6 million inhabitants in 2011, which is a 29% increase compared with 2001 [16]. Migration to the province is concentrated in the townships of the urban and suburban areas, where about 21% of the population of the province reside [16]. The living conditions there are precarious, characterized by crowding, and by the use of paraffin in up to 15% of households for heating, cooking, and lighting [16]. This, combined with the high number of young children, contribute to an increase in the risk for burn injuries to occur in and around the home, not least during winter. In fact, a peak in burn injuries during the winter season [17] has been observed in the Sub-Saharan region in general [18], and in the Western Cape in particular [14,19].

The RCH is the only public paediatric tertiary hospital in the whole Western Cape Province. The burns centre at RCH is the only one dedicated to burn patients under the age of 13 years in the province, and it can also take care of severely injured patients from the whole of Sub-Saharan Africa due to the lack of similar facilities in the entire region. The centre was modernized and upgraded in 2011 to improve patients’ outcomes and emotional support as well as to reduce cross-infection; it has 22 beds. Most admissions are preceded by a case assessment made by a specialist in the unit, whether patients were referred from another hospital or from the RCH trauma unit.

### 2.3. Data Collection

**Figure 1:**
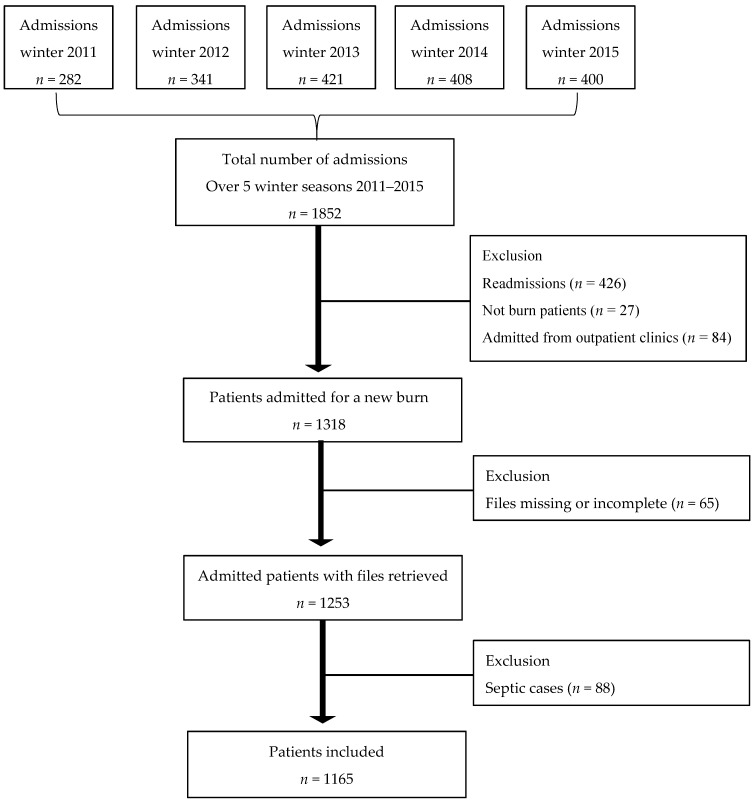
Flow chart of the inclusion/exclusion process used for data collection.

Data were collected for five consecutive winter seasons (1 May–15 August) when burn injuries peak, starting in 2011 when the burns centre was refurbished until 2015 when data collection took place. All patients admitted to the centre for acute burns during the reference periods were considered eligible. As our focus was on fresh burns, cases admitted from other surgical wards, those presenting with septic infected burn wounds, those admitted after several visits to the outpatient clinic, and those readmitted were excluded (Figure 1). As we expected, the number of admissions per annum increased slightly during the study period, with 916 admissions in 2011, 1016 in 2012, 1096 in 2013, 1102 in 2014, and 1133 in 2015.

Patients admitted were identified from the centre’s admission books using their hospital record number. Individual patient medical records were then retrieved from the hospital’s archives. A standardized electronic case report form covering a range of patient and injury characteristics was used for data collection purposes. All data used in this article can be found in Supplementary Table S1.

**Figure 2:**
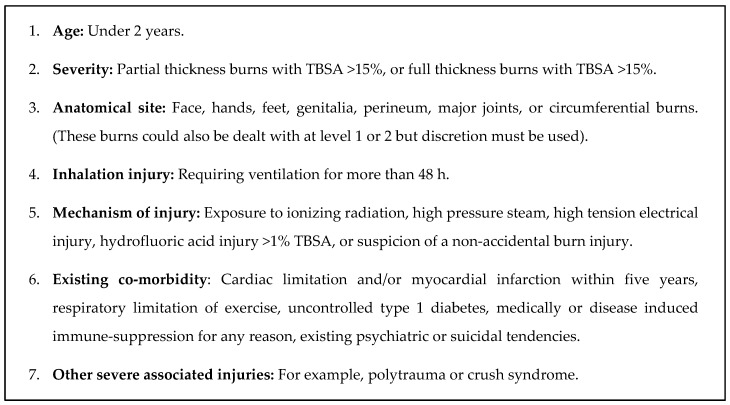
Western Cape provincial referral criteria for transfer to the paediatric burns centre [20].

The provincial referral guidelines currently in use were implemented in 2011 in the Western Cape Province [20] based on the South African Burn Society’s list from 2007 [12]. The seven referral criteria put forward in the case of paediatric patients (listed in Figure 2) include young age, burn severity, anatomical site injured, presence of an inhalation injury, mechanism of injury, existing co-morbidity, and presence of other severe associated injuries. Whether each one of the seven given criteria could be identified for a case was determined based on the information found in the case record. For any given case, several criteria could apply.

### 2.4. Data Analyses

A case was defined as adherent (i.e., admitted to the burns centre in accordance with the provincial referral guidelines) when at least one criterion was fulfilled. For any given group of cases, adherence to the referral guidelines was expressed as a percentage (number of cases where at least one criterion was fulfilled over the total number of cases × 100) and 95% confidence intervals were compiled.

Adherence was measured all years aggregated and for each of the five years (winter seasons) included in the study period. Changes in cases characteristics and adherence overtime were measured using an extension of the non-parametric Wilcoxon rank-sum test for trend, as described by Cuzick [21].

For patients aged 2 years and over, adherence was measured for each category of the following variables: gender (2 categories), age (3 categories), burn depth (2 categories), burn size (total body surface area (TBSA); 3 categories), and injury mechanism (3 categories).

We further assessed whether, when paediatric patients were admitted according to certain referral criteria, there was then an association between these and patient management. Patient management was expressed as a composite measure including the need for either surgery or a hospital length of stay longer than seven days. The analyses were stratified by age under and above 2 years. We conducted simple logistic regressions one criterion at a time, using as reference categories patients not fulfilling that criterion. The associations were expressed as odds ratios (OR) with 95% confidence intervals.

All data analyses were performed using Stata (version 13, College Station, TX, USA).

### 2.5. Ethical Approval

The study was conducted in accordance with the Declaration of Helsinki, and the protocol was approved by the Human Research Ethics Committee at Cape Town University (Dnr: 452/2015).

## 3. Results

Overall, during the five consecutive winter seasons, a total of 1165 admissions were identified and included. Table 1 summarizes the characteristics of those cases for all years aggregated and by year. There were more boys admitted than girls (boy to girl ratio: 1.3:1) and patients aged less than 2 years represented slightly more than half of the patients (51.9%) with a rapid decrease by age category thereafter. Most children were injured by hot liquids (scald burns) and a majority of the wounds were of partial thickness and with a TBSA ≤ 10%. The face was the site of injury in 42% of the cases. Further, for all years aggregated, 38% of the patients either stayed longer than seven days or underwent surgery. This rate was highest in 2013 and the lowest was in 2014. Trends analyses reveal that only TBSA significantly changed over time (z = −2.56; *p*-value = 0.01).

For each criterion listed in the guidelines, Table 2 presents the proportion of cases for which a given criterion could be identified given the data at hand in the patient records. Anatomical site was by far the most commonly identified criterion (85.2%), followed by age younger than 2 (51.9%). Within the anatomical site criterion, burns to the face were most frequently observed (42.1%), and burns to the genitalia were least often observed (9.6%). The severity criterion, defined as having a burn with TBSA > 15% was met by 11% of the patients overall, with 2.4% of the patients being identified as meeting the full thickness severity criterion. Finally, 6.6% of the patients were admitted without fulfilling any of the criteria.

On average, 1.7 criteria were identified in patients where at least one criterion could be identified. For a number of the patients (52%), 2 criteria were identified, and for 88 (8.3%) 3 criteria or more were identified. The most common combination of two criteria was that of anatomical site and age (45%), and any other criteria coincided less frequently with anatomical site or age (16.7% and 7.2%, respectively).

Table 3 presents the proportion of cases who were admitted in accordance with the referral guidelines (fulfilling at least one referral criterion), for all years aggregated and by year (winter seasons). Overall, adherence reached 93.4%. No significant differences (z = 0.13, *p*-value = 0.897) were found over the years.

When looking at the variation in adherence between different patient and injury characteristics, it appeared that it was maximal (100%) for children less than 2 years old, those with electrical or chemical burns, and those with a burn with a TBSA > 15%, as those categories are part of the criteria definitions. For those children that were 2 years and older, the adherence was 86.3%. In that age group, the adherence varied somewhat—but not significantly—depending on the child and injury characteristics considered, as shown in Table 4, from 79.2% in the case of TBSA 11−15% to 88.9% for contact burns with hot objects.

Overall, 443 patients (38.1%) either had to undergo surgery or had to stay longer than seven days at the centre. Adherence to the guidelines was not associated with having to undergo surgery or to stay longer than seven days (OR = 0.96; 95% CI: 0.60, 1.54; *p*-value = 0.866). Yet, patients ≥2 years of age, who did not fulfil the age criterion, were significantly more likely to have to undergo surgery or to have to stay longer than those who did (OR = 1.76; 95% CI: 1.39, 2.23; *p*-value < 0.001). Table 5 presents, for each criterion, the odds of a patient having to undergo surgery or to stay longer than seven days when fulfilling the criterion compared to not fulfilling it for patients younger and those at least 2 years of age. For children aged under 2 years, only the severity criterion was significantly associated with having to undergo surgery or to stay for a long length of stay (OR = 19.4). For all the other criteria there was no association between patient management and fulfilment of the criteria. In the older age group, the odds of having surgery or of staying longer than seven days at the centre was 11.6 times higher for patients fulfilling the severity criterion compared to those that did not. Similarly, patients with an inhalation injury were more likely to have a complex management than patients not fulfilling that criterion (OR = 5.5), which was not the case for patients aged <2 years.

## 4. Discussion

The results reveal that adherence to the provincial referral guidelines at the centre is very high, over 93%, for all five winter seasons aggregated as well as for any year. It is maximal (100%) for children under 2 years of age (a referral criterion) and around 86% for children aged 2 years and older, with some small variations depending on the patient or injury characteristic taken into account.

The referral criteria most often identified in the patient records were, by far, anatomical site injured and young age. As a group, children two years and older were more likely to undergo surgery or stay longer than seven days compared to their younger counterparts. However, when considering admissions based on the injury severity as a criterion, in both age groups those meeting the criterion were much more likely to undergo surgery or to stay longer than seven days at the centre compared to those who did not.

Studies on adherence to referral criteria at burn centres, be it for adults or children, are very few. To the best of our knowledge, the level of adherence found in this study is the highest reported in the scientific literature for a burns centre, compared to that of a centre in the United States (88% for adults [22], and 76% for children [23]) and one in Denmark (70% for all ages) [8]. Whether this is a reflection of differences between the catchment area, the case characteristics, the healthcare systems, or the referral criteria in place (in kind and number) remains to be determined. There is no published data on the demographics of the paediatric patients at the US burns centre [23], but there could be some differences judging from differences reported between the caseload of the RCH and that of a burns centre in Scotland [19]. In addition, we know for a fact that the nature [8] and the number of referral criteria evaluated [22,23] differ between the different publications. In the US studies, for instance, only two criteria were used, representing only burns to specific anatomical parts and of a specific severity [22,23].

It comes a bit as a surprise that severity as a criterion applied to only 11% of the cases. This value is much lower than that reported at the Danish centre (56%; all ages) [8] and at the US centre (33% for paediatric patients) [23]. However, the criterion definition is not the same. Had we used the TBSA cut-offs from those other centres (TBSA > 3% in Denmark [8] and TBSA > 10% in the US [23]), it would have translated to either 78% or 28% of the RCH patients. Why the centres actually use different severity criteria is unclear. The same applies to anatomical sites [8,23].

It is also of note that the South African guidelines include young age as a referral criterion, for which the adherence was found to be at 100%, but the two other centres do not [8,9]. Incidentally, in the Danish centre, over-triage is most common among toddlers [8].

Our findings relative to patient care in light of the criterion of admission that was identified are not easy to compare, as this has not been studied previously. In the Danish study, all patients who underwent surgery were adherent cases [8]. However, our results find an echo in the results from an Australian burns centre, where admission due to burn severity was associated with both longer length of stay and higher surgery rates [24]. In addition, according to the authors of a previous study from a burns centre in Kwa-Zulu Natal (South Africa), the high number of “inappropriate referrals” in their centre was associated with the low rates of 17% of children and 16% of adults that actually required tangential excision performed by burn specialists [25].

That adherence to the referral guidelines at the centre is high is of course positive. Still, it should be emphasized that the average rate of non-adherence of 6.6% translates to one out of the 22 beds being used by a patient during the winter seasons that did not fulfil any of the referral criteria. The implications of this potential over-triage in a resource-constrained centre where the burden of burns is high can be substantial. Indeed, a recent study performed at the trauma unit from the same hospital shows that there was a high number of patients who fulfilled the referral guidelines but who were discharged from the unit and not transferred to the burns centre [13]. From our own data we also know that, for all years aggregated, as many as 88 children with septic burns were admitted to the unit (about 17 per annum and winter), which could suggest some flaws in the assessment of the severity of the burn—or condition of the patient—in the acute phase.

This study is one of the very few that investigate how patient admissions to a burns centre comply with the referral criteria in place [8,22,23], and it is the first one in a low- and middle-income setting. Several consecutive years are taken into consideration and this allows for an assessment of the relative variability of the information. It is well known that there are seasonal variations in the prevalence of paediatric burns in South Africa [14,17,26]. By choosing the winter as a point of observation over time, we focused on the one season where the demand for burn care is expected to peak. Whereas it seems that admission follows the criteria in use, the study is unable to comment as to whether there are seasonal variations in that respect.

Still related to the coverage, no patients were identified that matched the referral criterion “severe other associated injuries”. Those cases might have been admitted into other wards without being registered at the burns centre. Similarly, no data on deceased patients could be included. Having included them would most likely have increased the adherence level.

Concerning the anatomical site criterion, the guidelines explicitly state that patients could possibly be treated at lower levels of care [20]. The study does not tell whether this could have been the case or not, and the impact of this eventuality on the results obtained is hard to determine.

The study also does not clarify the actual reason for admission—rather, the results are based on information extracted from the patients’ medical records where referral criteria were sought. As a consequence, we do not know whether some criteria matter more than others in decisions relative to admission. We do know, however, that two criteria from the guidelines are observed far more than others—anatomical site and young age—in the patient files.

Although patient care here was assessed based on surgery and length of stay, admission to a burns centre is not exclusively based on the need for surgery: the overall need of specialized care, which can include but is not limited to surgery, is most likely to influence the final decision [14].

Even though the level of over-triage at the centre was low, it has previously been reported that a number of patients at primary and secondary care [14] as well as at the trauma unit [13] meet at least one criterion. The study is also silent as regards whether children who are not transferred or not admitted to the centre suffer increased complications, such as septic wounds, or suffer from additional comorbidities. Indeed, given the high incidence of burn injuries in the region, it is likely that there is some under-triage at lower levels of care.

## 5. Conclusions

In this specialized paediatric burns centre of the Red Cross War Memorial Hospital, children are admitted mainly according to the referral guidelines. Anatomical site injured and young age are apparently the most common criteria identified. While children aged 2 years and older received more care in terms of surgery or duration of stay than those of a young age, those fulfilling the severity criterion also received more surgery or stayed longer at the centre. However, given the relatively small size of the centre and the high prevalence of paediatric burns in the region, adherence to the referral guidelines in place may need to be further studied at all healthcare levels of the province.

## Figures and Tables

**Table 1:** Description of the characteristics of the cases, including patient, injury, and treatment, for all years aggregated and by year of admission.

Case Characteristics	All Years Aggregated 2011–2015	2011	2012	2013	2014	2015
	*n* = 1165	*n* = 166	*n* = 208	*n* = 282	*n* = 259	*n* = 250
	%	%	%	%	%	%
**Patient gender**						
Boys	55.7	57.2	54.8	56.0	54.1	56.8
Girls	44.3	42.8	45.2	44.0	45.9	43.2
**Patient age**						
Infant/toddler (<2 years)	51.9	51.2	51.9	55.7	51.4	48.4
Late toddler (2–3 years)	26.8	24.1	26.0	25.5	25.1	32.4
Preschool (4–5 years)	9.1	9.6	10.6	8.5	10.8	6.4
School age (6–12 years)	12.3	15.1	11.5	10.3	12.7	12.8
**Injury mechanism**						
Hot liquid	81.8	80.1	76.4	85.1	83.4	82.0
Hot object	6.7	6.0	10.1	5.0	4.3	8.8
Fire/Flame	9.3	9.6	11.5	7.1	10.8	8.0
Electrical or Chemical	2.0	3.6	1.9	2.5	1.2	1.2
Unknown	0.3	0.6	0.0	0.4	0.4	0.0
**Burn depth**						
Partial thickness	88.9	87.4	82.7	92.9	92.3	87.3
Full thickness	8.8	11.5	13.5	4.6	5.8	10.8
Unknown	2.3	1.2	3.9	2.5	1.9	2.0
**TBSA (in %)**						
≤5	40.5	28.3	44.7	37.2	49.4	39.6
6–10	31.9	34.9	29.8	30.9	31.3	33.6
11–15	15.7	20.5	14.4	20.2	12.4	12.0
16–20	5.6	7.8	5.3	5.0	3.9	6.8
≥21	5.5	8.4	3.9	5.7	3.1	7.2
Unknown	0.8	0.0	1.9	1.1	0.0	0.8
**Anatomical site**						
None of the criterion-specified anatomical sites	14.9	11.5	14.9	16.7	17.8	12.0
Criterion-specified anatomical sites	85.2	88.6	85.1	83.3	82.2	88.0
Face ^1^	42.1	46.4	39.9	40.1	44.0	41.2
Extremities (hands or feet) ^1^	32.2	30.1	33.7	28.0	32.1	37.2
Genitalia or perineum ^1^	9.6	13.9	8.7	6.7	7.3	13.2
Major joint or circumferential ^1^	31.7	31.9	29.3	41.1	24.3	30.4
**Patient care**						
Length of stay longer than seven days or need of surgery	38.1	39.8	35.1	42.9	34.1	38.0
Length of stay shorter than seven days and no surgery	61.9	60.2	64.9	57.1	65.9	62.0

^1^ The categories are not mutually exclusive.

**Table 2:** Proportion of cases for which a given criterion was identified (*n* = 1165).

Criteria	Cases in Which the Criterion Was Identified		
	*n*	%	95% CI
Anatomical site	992	85.2	(83.0, 87.1)
Age	604	51.9	(48.9, 54.7)
Severity	129	11.1	(9.3, 13.0)
Inhalation injury	33	2.8	(2.0, 4.0)
Mechanism of injury	45	3.9	(2.8, 5.1)
Existing co-morbidity	35	3.0	(2.1, 4.2)
Severe other injuries	0	0.0	-
None of the referral criteria	77	6.6	(5.3, 8.2)

**Table 3:** All years aggregated and yearly adherence to the referral guidelines.

Winter of Admission		Adherence		
Year	N	*n*	%	95% CI
All years aggregated 2011–2015	1165	1088	93.4	(91.8, 94.7)
2011	166	155	93.4	(88.5, 96.6)
2012	208	195	93.8	(89.5, 96.6)
2013	282	264	93.6	(90.1, 96.2)
2014	259	237	91.5	(87.4, 94.6)
2015	250	237	94.8	(91.3, 97.2)

**Table 4:** Adherence to the referral guidelines by patient and burn characteristics for children ≥2 years of age (*n* = 561).

Characteristic		Adherence		
	N	*n*	%	95% CI
**Gender**				
Boys	311	275	88.4	(84.3, 91.8)
Girls	250	209	83.6	(78.4, 88.0)
**Age (in years)**				
2–3	312	267	85.6	(81.7, 89.5)
4–5	106	91	85.9	(79.1, 92.6)
6–12	143	126	88.1	(82.7, 93.5)
**Burn depth**				
Partial thickness	477	414	86.8	(83.4, 89.7)
Full thickness	72	58	80.6	(69.5, 88.9)
**TBSA (in %)**				
≤5	202	179	88.6	(83.4, 92.6)
6–10	174	142	81.6	(75.0, 87.1)
11–15	106	84	79.2	(20.3, 86.5)
**Mechanism**				
Hot liquid	419	359	85.7	(82.0, 88.9)
Fire/Flame	84	71	84.5	(75.0, 91.5)
Hot object	36	32	88.9	(73.9, 96.9)

**Table 5:** Association between single referral criteria and burden of having to undergo surgery or stay longer than seven days at the centre by age category of the child.

Criteria	Children Aged < 2 Years			Children Aged ≥ 2 Years		
	OR	95% CI	*p* Value	OR	95% CI	*p* Value
Anatomical site	1.4	(0.8, 2.3)	0.256	1.2	(0.7, 1.8)	0.526
Severity	19.4	(8.6, 43.9)	<0.001	11.6	(5.6, 23.8)	<0.001
Inhalation injury	2.2	(0.7, 6.9)	0.184	5.5	(1.8, 16.6)	0.001
Mechanism of injury	2.7	(0.7, 10.3)	0.139	1.1	(0.6, 2.2)	0.765
Existing co-morbidity	1.8	(0.7, 4.5)	0.250	0.5	(0.2, 1.4)	0.183

## Supplementary Materials

The following are available online at www.mdpi.com/1660-4601/14/7/732/s1, Table S1: raw data material used for this study.
